# Impact of the COVID-19 Pandemic on Healthcare-Seeking Behaviors among Frequent Emergency Department Users: A Cohort Study

**DOI:** 10.3390/ijerph18126351

**Published:** 2021-06-11

**Authors:** Yi-Chang Chou, Yung-Feng Yen, Dachen Chu, Hsiao-Yun Hu

**Affiliations:** 1 Department of Education and Research, Taipei City Hospital, Taipei 106, Taiwan; T0036@tpech.gov.tw (Y.-C.C.); DAM37@tpech.gov.tw (Y.-F.Y.); 2 Institute of Public Health, National Yang Ming Chiao Tung University, Taipei 112, Taiwan; 3 Section of Infectious Diseases, Taipei City Hospital, Yangming Branch, Taipei 111, Taiwan; 4 Department of Health Care Management, National Taipei University of Nursing and Health Sciences, Taipei 112, Taiwan; 5 Department of Psychology and Counseling, University of Taipei, Taipei 100, Taiwan; 6 Department of Health and Welfare, University of Taipei, Taipei 100, Taiwan; dad57@tpech.gov.tw; 7 Institute of Hospital and Health Care Administration, National Yang Ming Chiao Tung University, Taipei 112, Taiwan; 8 Department of Neurosurgery, Taipei City Hospital, Taipei 103, Taiwan

**Keywords:** emergency department, frequent users, COVID-19 pandemic

## Abstract

In 2020, Taiwan’s healthcare system faced a notable burden imposed by the coronavirus disease (COVID-19) pandemic. Emergency department (ED) is a high-risk area for severe acute respiratory syndrome coronavirus 2 transmission. The effect of COVID-19 on the utilization of ED services among frequent ED users remains unknown. This cohort study determined the impact of the COVID-19 pandemic on healthcare-seeking behaviors among frequent ED users at Taipei City Hospital, Taiwan. We included ED users aged ≥ 18 years admitted to Taipei City Hospital during February 2019–January 2020 (before the pandemic) and February 2020–January 2021 (during the pandemic). Frequent ED users were patients with four or more ED visits per year. Stepwise logistic regression was performed to identify predictors of frequent ED use during the COVID-19 pandemic. Frequent ED users had shorter hospital stays in the ED during the pandemic. After adjusting for sociodemographic factors and other covariates, patients with a triage status of level 4–5, pneumonia diagnosis, giddiness, or dyspnea were more likely frequent ED visitors during the COVID-19 pandemic. To reduce the risk of acquiring COVID-19, it is important to utilize territorial healthcare or telehealth to avoid inappropriate ED visits for patients with a low level of risk or chronic disease.

## 1. Introduction

Emergency department (ED) crowding is a burden on public health [1,2], so understanding the characteristics of frequent ED users is a key concern of healthcare systems and policy makers [3,4]. Taiwan’s implementation of National Health Insurance in 1994 enhanced public access to healthcare. From 2000 to 2015, the number of ED visits in Taiwan increased by about 20.7%, leading to ED crowding and a larger number of frequent ED users [5,6].

As compared to occasional ED users, frequent ED users are older [7], have more chronic diseases [8], complex mental health problems [9,10], or drug addiction [11,12,13]. It has been shown that it is possible to reduce the number of visits by frequent ED users through certain intervention measures, such as case management, personal nursing care planning, strategies for pre-hospital transfer to non-emergency care, and enhanced primary care [14,15]. Therefore, identifying the features of frequent ED users and designing appropriate intervention measures are crucial tasks for reducing the frequency of ED visits and improving the relevant healthcare outcomes. 

In 2020, Taiwan’s healthcare system faced the significant challenge posed by the coronavirus disease (COVID-19) pandemic, and emergency care became the front-line tactic in the battle against this disease. To reduce disease transmission, the US Centers for Disease Control and Prevention (CDC) issued stay-at-home recommendations and encouraged local governments or healthcare systems to adopt corresponding policies or healthcare regulations.

The infectious nature of COVID-19 can influence patients to avoid visiting the hospital due to fears surrounding the rapid transmissibility of the disease. This restriction subsequently prevents patients from seeking medical care, which may decrease the utilization of ED services. A recent study conducted in the US showed that there was a 49.3% decline in ED visits after the declaration of the COVID-19 pandemic [16]. Another study conducted in Germany demonstrated a drop of 63.8% in pediatric emergency healthcare utilization during the COVID-19 pandemic [17]. Although recent studies have indicated a negative impact of the COVID-19 pandemic on the utilization of ED services, there has been scarce evidence for the impact of the COVID-19 pandemic on the utilization of ED services among frequent ED users.

The present study aimed to determine the impact of the COVID-19 pandemic on healthcare-seeking behaviors among frequent ED users at Taipei City Hospital (TCH), Taiwan. In addition, this study identified predictors associated with frequent ED use before and during the COVID-19 pandemic.

## 2. Materials and Methods

### 2.1. Study Design and Setting

TCH, a 4700-bed hospital, is the largest healthcare organization in northern Taiwan. This cohort study examined patients who visited TCH Renai, Heping, and Zhongxiao branches in February 2019–January 2021. All information that could identify a specific individual patient was encrypted. After data encryption, emergency department data, medical utilization, discharge data, and demographic information was collected. The data used in the study were de-identified before the analysis took place. The study protocol was approved by the institutional review board (TCHIRB-10904009-E).

### 2.2. Selection of Participants

**Figure 1:**
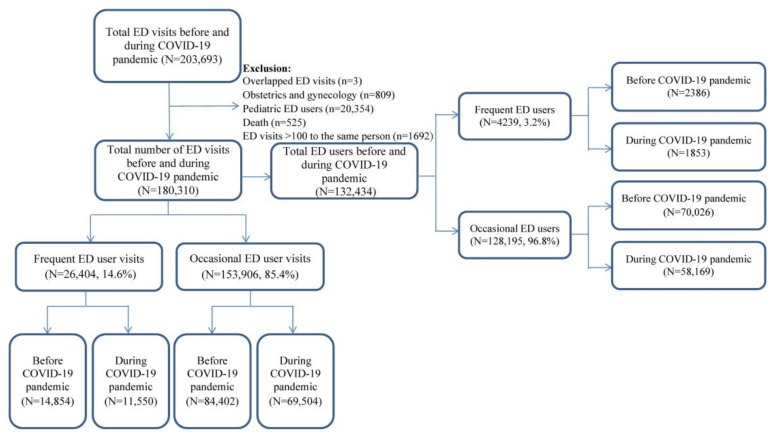
Flow chart of the patient selection process.

This cohort study compared the utilization of emergency medical services before (February 2019–January 2020) and during (February 2020–January 2021) the COVID-19 pandemic. Individuals aged ≥ 18 years who visited the TCH ED during these two periods were included as participants. As this study intended to determine the impact of the COVID-19 pandemic on healthcare-seeking behaviors among adult frequent ED users, this study excluded the following types of patients from the analysis: pediatric ED users (*n* = 20,354), pregnant individuals (*n* = 809), and patients with out-of-hospital cardiac arrest deaths or death after an ED visit (*n* = 525) (Figure 1).

### 2.3. Measurements 

Covariates identified as predictors [18] of frequent visit to the ED in previous studies were assessed in our analyses; these included the individuals’ sociodemographic characteristics (sex, age, and copayment exemptions), triage status, mode of patient arrival, and most frequent primary diagnoses during ED visits. All medical records in the periods before and during the COVID-19 pandemic were collected. Subjects were classified into age groups of 18–34 years, 35–49 years, 50–64 years, 65–79 years, and ≥80 years. Most patients with copayment exemptions had catastrophic illnesses and were exempt from paying approximately US $10 in medical expenses [19]. The characteristics of ED visit included the time of ED visit (8 a.m.–4 p.m., 4 p.m.–0 a.m., 0 a.m.–8 a.m.), disposition of ED visit (discharge, hospitalization), triage status of patient severity (levels 1–5) [20], mode of patient arrival (walk-in, referral from other institutions, ambulance), average length of stay (LOS) in the ED, average medical expenses, cancer, chest radiography, and chest computed tomography (CT) examination. Data of the ten most frequent primary diagnoses in ED visits were also collected for the periods before and during the COVID-19 pandemic.

### 2.4. Outcomes

The outcome variable was the frequency of ED user visits before and during the COVID-19 pandemic. Frequent ED users were defined as those with four or more ED visits in a year, and occasional ED users were defined as those with one to three ED visits [2,10,21].

### 2.5. Data Analysis

Analyses were performed on the personal characteristics, healthcare utilization characteristics, and the top ten primary diagnoses before and during the COVID-19 pandemic for frequent and occasional ED users. The data were presented as percentages, and a chi-squared test or Student’s *t*-test was performed to compare the differences between the periods before and during the pandemic. The predictors for frequent ED use were analyzed using multivariate logistic regression, and the forward stepwise regression model was adopted, adjusting for age, sex, triage status, mode of patient arrival, copayment exemption status, and top ten primary diagnoses of the given year. Statistical significance was set at 5%, and all analyses were conducted using SAS (version 9.4; SAS Institute, Inc., Cary, NC, USA).

## 3. Results

### 3.1. Characteristics of Study Subjects

In total, 132,434 patients who visited the TCH ED before and during the pandemic were included in this study. The number of ED users significantly decreased from 72,412 before the COVID-19 pandemic to 60,022 during the COVID-19 pandemic (*p* = 0.032). The overall mean (standard deviation) age was 49.6 (21.0) years, and 49.2% of the participants were male. Of the 132,434 patients, 12,423 (9.4%) had medical records for both periods. Frequent ED users accounted for 3.3% (2386 cases) and 3.1% (1853 cases) of ED patients before and during the COVID-19 pandemic, respectively (Table 1).

### 3.2. Trend of Monthly Emergency Department Visits before and during COVID-19 Pandemic

**Figure 2:**
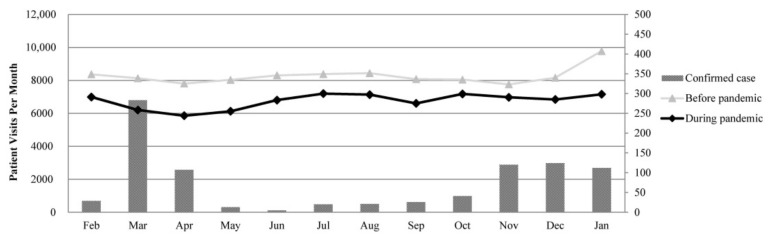
Trend of monthly emergency department visits before and during the COVID-19 pandemic.

Figure 2 shows the trend of monthly ED visits before and during the COVID-19 pandemic. After the start of the COVID-19 outbreak in January 2020, the number of ED visits during the COVID-19 pandemic significantly decreased by 10.1–26.8% compared to that before the COVID-19 pandemic (*p* < 0.0001). During the COVID-19 pandemic, the number of laboratory-confirmed COVID-19 cases in Taiwan significantly decreased from 283 cases in March to 20 cases in July. The number of monthly ED visits during the COVID-19 pandemic slightly increased from 6202 ED visits in March to 7196 ED visits in July (*p* = 0.004), which, however, was lower than before the COVID-19 pandemic.

### 3.3. Characteristics of ED Visits before and during COVID-19 Pandemic

A total of 180,310 ED visits were recorded during the study period, including 99,256 (55.1%) and 81,054 (44.9%) before and during the COVID-19 pandemic, respectively. In terms of frequent ED users, that is those visiting the ED four times or more, these patients had a shorter length of stay in the ED compared to before the COVID-19 pandemic (212.5 vs. 233.9 min; *p* < 0.0001) (Table 2).

### 3.4. Primary Diagnoses in ED Users before and during COVID-19 Pandemic

The top ten most frequent primary diagnoses in ED users were analyzed before and during the COVID-19 pandemic (Appendix A, Table A1). Among frequent ED users, the most frequent primary diagnoses during the COVID-19 pandemic were dizziness and giddiness (5.71%), followed by abdominal and pelvic pain (5.68%), and fever of unknown origin (3.55%). Moreover, the top three primary diagnoses among frequent ED users before the COVID-19 pandemic were abdominal and pelvic pain (5.75%), dizziness and giddiness (5.04%), and fever of unknown origin (4.10%).

### 3.5. Factors Associated with Frequent ED Users before and during COVID-19 Pandemic

Table 3 shows the multivariate analyses for factors associated with frequent ED users before and during the COVID-19 pandemic. After adjusting for sociodemographic factors and other covariates, patients with a triage status of level 4–5 (AOR = 1.63, 95% CI: 1.15–2.31), a diagnosis of pneumonia (AOR = 1.88, 95% CI: 1.09–3.24), dizziness and giddiness (AOR = 2.88, 95% CI: 1.84–4.52), dyspnea (AOR = 1.83, 95% CI: 0.99–3.37), or chronic kidney disease (AOR = 5.07, 95% CI: 2.66–9.69) were more likely to visit the ED four or more times during the COVID-19 pandemic. Moreover, patients aged ≥ 65 years, those with copayment exemptions, and those with cancer were more likely to visit the ED four or more times before and during the COVID-19 pandemic.

## 4. Discussion

This study found that the utilization of emergency medical services during the COVID-19 pandemic significantly decreased by 10.1–26.8% compared to before the COVID-19 pandemic. The LOS in frequent ED users during the COVID-19 pandemic was significantly shorter than that before the COVID-19 pandemic. Furthermore, patients with a triage status of level 4–5, or a diagnosis of pneumonia, giddiness, or dyspnea were more likely to frequently utilize the emergency medical services during the COVID-19 pandemic.

This cohort study showed that the overall ED service volume during the COVID-19 pandemic in Taiwan decreased significantly by 26.8%, which was lower than the reductions of 39.6% seen in EDs in the US [22] and 63.8% in the pediatric ED in Germany [17]. The relatively lower impact of the COVID-19 pandemic on the utilization of ED services in Taiwan may be due to the successful control of the COVID-19 pandemic in 2020. As the COVID-19 outbreak emerged, the Taiwanese government implemented several strategies to prevent the nationwide spread of COVID-19, including border controls, proactive screening measures, and quarantine procedures [23,24]. By 31 January 2021, 911 laboratory-confirmed COVID-19 cases were reported to the Taiwan Centers for Disease Control and Prevention (CDC), including 797 (87.5%) imported cases [25]. Although Taiwan successfully controlled the spread of the COVID-19 pandemic in the country in 2020, there was still a significant overall reduction in ED utilization. As ED services provide treatments for patients with acute illnesses, it is important to raise patient awareness regarding acute health conditions that are deadlier than COVID-19 and that require immediate medical intervention to ensure health and recovery.

With regards to the utilization of ED services, the average LOS of frequent ED users showed a significant decrease of 21.4 min (*p* < 0.0001). This could be attributed to the COVID-19 pandemic, with physicians reducing observation times in order to avoid the risk of nosocomial infections. A previous study in Canada showed that the length of stay in ED users was significantly decreased in a pediatric emergency department during the SARS pandemic of 2003 [26]. Patients staying in the ED for longer periods of time during the COVID-19 pandemic increases the risk of a SARS-CoV-2 outbreak in crowded ED departments. The findings of our study suggest that it is important to reduce the LOS of ED users to prevent the occurrence of SARS-CoV-2 infection in these patients.

This study found that patients with a diagnosis of pneumonia were more likely to utilize emergency medical services frequently during the COVID-19 pandemic. The implementation of enhanced traffic control bundling (eTCB) to prevent COVID-19 outbreaks in Taiwan may explain the high frequency of emergency medical service use in patients with a diagnosis of pneumonia. In the beginning of the COVID-19 epidemic in 2020, Taiwan CDC implemented eTCB in nationwide hospitals to secure the healthcare system [27]. At the hospital entrance, all patients were required to undergo body temperature and TOCC (i.e., travel history, occupation, contact history, and clusters) checks before entering. If patients at the hospital entrance presented with fever, symptoms of pneumonia, or a history of visiting regions with a declared COVID-19 outbreak during the last 14 days, they were referred to the ED for a COVID-19 examination. Since healthcare workers are vulnerable to SARS-CoV-2 infection, the urgent adoption of strict COVID-19 prevention strategies was essential to prevent COVID-19 outbreaks in healthcare settings.

This study showed that patients with triage status of level 4–5 or the symptoms of dizziness or giddiness were more likely to frequently utilize ED medical services during the COVID-19 pandemic. The increasing burden of COVID-19-related psychological disorders may explain the high frequency of the utilization of emergency medical services in patients with a triage status of level 4–5 or the symptoms of dizziness or giddiness. Recent reports have shown that the COVID-19 pandemic has increased the burden of mental and psychological problems in the general public [28,29,30], which could increase the utilization of emergency medical services. Since SARS-CoV-2 is highly contagious, it is imperative to educate non-emergency patients to utilize outpatient medical services rather than emergency medical services to reduce the risk of COVID-19 infection and outbreaks at ED.

There were two limitations to this study. First, the data for ED visits originated from a single hospital, which did not include all ED visits in the entire region. However, due to the COVID-19 pandemic, people have reduced their use of public transport and opted for hospitals closer to their homes, in order to avoid the risk of infection, while also cutting down on unnecessary visits. Therefore, cases where patients visit different hospitals for the same disease were expected to have decreased. Second, although TCH is the largest healthcare organization in northern Taiwan, our subjects were selected only from a single hospital. Therefore, the external validity of our findings may be of concern, and the generalizability of our results to hospital settings other than non-Asian ethnic groups requires further verification.

## 5. Conclusions

This cohort study demonstrated that the utilization of emergency medical services was significantly decreased during the COVID-19 pandemic. Patients with a triage status of level 4–5, a pneumonia diagnosis, giddiness, or dyspnea were more likely to frequently utilize the emergency medical services during the COVID-19 pandemic. To reduce the risk of SARS-CoV-2 infection transmission, it is important to utilize territorial healthcare or telehealth to avoid inappropriate ED visits for patients with a low level of risk or with chronic disease.

## Figures and Tables

**Table 1:** Characteristics of ED patients before (February 2019–January 2020) and during COVID-19 pandemic (February 2020–January 2021) *(n* = 132,434).

	Frequent ED Users		*p*-Value	Occasional ED Users		*p*-Value
n (%)	Before	During		Before	During	
Total	2386 (3.3)	1853 (3.1)		70,026 (96.7)	58,169 (96.9)	0.032
Sex						
Male	1276 (53.5)	1016 (54.8)	0.381	33,932 (48.5)	28,938 (49.8)	<0.0001
Female	1110 (46.5)	837 (45.2)		36,094 (51.5)	29,231 (50.2)	
Age, y (Mean ± SD)	65.3 ± 20.7	65.0 ± 20.7	0.605	50.0 ± 21.0	49.9 ± 21.3	0.347
18–34	249 (10.4)	199 (10.7)	0.247	20,979 (30.0)	18,233 (31.3)	<0.0001
35–49	325 (13.6)	252 (13.6)		14,836 (21.2)	11,877 (20.4)	
50–64	487 (20.4)	365 (19.7)		14,818 (21.2)	11,631 (20.0)	
65–79	556 (23.3)	483 (26.1)		11,906 (17.0)	9788 (16.8)	
>80	769 (32.2)	554 (29.9)		7487 (10.7)	6640 (11.4)	
Copayment exemptions						
Yes	852 (35.7)	696 (37.6)	0.214	7977 (11.4)	7000 (12.0)	<0.0001
No	1534 (64.3)	1157 (62.4)		62,049 (88.6)	51,169 (88.0)	
ED, Emergency Department.						

**Table 2:** Characteristics of ED visits before (February 2019–January 2020) and during the COVID-19 pandemic (February 2020–January 2021) *(n* = 180,310).

	Frequent ED Users		*p*-Value	Occasional ED Users		*p*-Value
N(%)	Before	During		Before	During	
Total	14,854 (15.0)	11,550 (14.2)		84,402 (85.0)	69,504 (85.8)	
Time of visits						
Daytime (8 a.m.–4 p.m.)	6534 (44.0)	5129 (44.4)	0.737	34,611 (41.0)	28,858 (41.5)	0.010
Evening (4 p.m.–0 a.m.)	5674 (38.2)	4398 (38.1)		35,868 (42.5)	29,366 (42.3)	
Early morning (0 a.m.–8 a.m.)	2646 (17.8)	2023 (17.5)		13,923 (16.5)	11,280 (16.2)	
Disposition						
Discharged	11,983 (80.7)	9246 (80.1)	0.208	72,265 (85.6)	58,763 (84.5)	<0.0001
Hospitalization	2871 (19.3)	2304 (19.9)		12,137 (14.4)	10,741 (15.5)	
Triage status						
1 (high)	379 (2.5)	309 (2.7)	0.006	1516 (1.8)	1472 (2.1)	<0.0001
2	1590 (10.7)	1247 (10.8)		6760 (8.0)	5401 (7.8)	
3	8034 (54.1)	6468 (56.0)		53,529 (63.4)	44,505 (64.0)	
4	3945 (26.6)	2882 (24.9)		20,551 (24.4)	15,559 (22.4)	
5 (low)	906 (6.1)	644 (5.6)		2046 (2.4)	2567 (3.7)	
Mode of arrival						
Ambulatory	10,734 (72.3)	8712 (75.5)	<0.0001	61,118 (72.4)	51,932 (74.8)	<0.0001
EMS	2560 (17.2)	2268 (19.7)		15,397 (18.2)	14,127 (20.3)	
Referral	1560 (10.5)	552 (4.8)		7887 (9.3)	3381 (4.9)	
Cancer			0.150			0.300
No	2232 (93.6)	1753 (94.6)		69,412 (99.1)	57,627 (99.1)	
Yes	154 (6.4)	100 (5.4)		614 (0.9)	542 (0.9)	
Over 24-h LOS	2259 (15.2)	1831 (15.9)	0.151	12,908 (15.3)	11,156 (16.1)	<0.0001
Length of stay (minutes) (Mean ± SD)	233.9 ± 366.1	212.5 ± 320.5	<0.0001	156.7 ± 268.3	154.4 ± 239.7	0.068
Medical expenses* (USD) (Mean ± SD)	108.4 ± 109.7	117.6 ± 111.0	<0.0001	102.1 ± 115.9	113.8 ± 110.0	<0.0001
Chest X-ray	5768 (38.8)	4628 (40.1)	0.041	28,332 (33.6)	26,670 (38.4)	<0.0001
Chest CT examination	72 (0.5)	120 (1.0)	<0.0001	513 (0.6)	806 (1.2)	<0.0001

ED, emergency department; EMS, emergency medical services; LOS, length of stay. * Medical expenses are presented in US dollars. (US dollars: NT dollars = 1:28.14). 12 March 2021.

**Table 3:** Factors associated with frequent ED users before (February 2019–January 2020) and during the COVID-19 pandemic (February 2020–January 2021).

Before the COVID-19 Pandemic			During the COVID-19 Pandemic		
Independent Variables	Adjusted OR (95% CI)	*p*-Value	Independent Variables	Adjusted OR (95% CI)	*p*-Value
Age ≥ 65	2.94 (2.48–3.48)	<0.0001	Age ≥ 65	2.89 (2.38–3.50)	<0.0001
Time of visits, evening (1600–2400)	0.80 (0.68–0.95)	0.009	Time of visits, early morning (0000–0800)	1.38 (1.08–1.77)	0.011
Mode of arrival, EMS	0.56 (0.43–0.72)	<0.0001	Mode of arrival, EMS	0.73 (0.56–0.95)	0.019
Copayment exemptions	3.94 (3.31–4.68)	<0.0001	Copayment exemptions	3.76 (3.10–4.56)	<0.0001
Primary diagnosis in the ED			Triage status, 4–5	1.63 (1.15–2.31)	0.006
Other anemias	4.55 (2.54–8.16)	<0.0001	Primary diagnosis in the ED		
Retention of urine	3.26 (1.69–6.28)	<0.0001	Dizziness and giddiness	2.88 (1.84–4.52)	<0.0001
Pain in throat and chest	1.88 (1.13-3.12)	0.015	Other anemias	5.14 (3.16–8.37)	<0.0001
Dizziness and giddiness	1.67 (1.01–2.77)	0.047	Dyspnea	1.83 (0.99–3.37)	0.053
Comorbidity of cancer	5.69 (3.81–8.50)	<0.0001	Retention of urine	5.56 (3.05–10.1)	<0.0001
			Pneumonia, unspecified organism	1.88 (1.09–3.24)	0.023
			Chronic kidney disease	5.07 (2.66–9.69)	<0.0001
			Comorbidity of cancer	3.41 (2.05–5.58)	<0.0001

ED, emergency department; CI, confidence interval; OR, odds ratio. Adjusted for age, sex, time of visits, triage status, mode of arrival, copayment exemptions, and the most frequent primary diagnoses among ED users before and during the COVID-19 pandemic.

## Data Availability

The datasets produced and analyzed during the present study are available from the corresponding author upon reasonable request.

## Appendix A

**Table A1:** The ten most frequent primary diagnoses among frequent and occasional ED users before (February 2019–January 2020) and during the COVID-19 pandemic (February 2020–January 2021) (*n* = 180,310).

Frequent ED Users (*n* = 26,404)			
Before the COVID-19 Pandemic (*n* = 14,854)	N (%)	During the COVID-19 Pandemic (*n* = 11,550)	N (%)
Abdominal and pelvic pain	855 (5.75)	Dizziness and giddiness	660 (5.71)
Dizziness and giddiness	749 (5.04)	Abdominal and pelvic pain	657 (5.68)
Fever of other and unknown origin	610 (4.10)	Fever of other and unknown origin	411 (3.55)
Other anemias	461 (3.10)	Other anemias	400 (3.46)
Pneumonia, unspecified organism	437 (2.94)	Pain in throat and chest	391 (3.38)
Retention of urine	379 (2.55)	Dsypnea	330 (2.85)
Pain in throat and chest	377 (2.53)	Other disorders of urinary system	312 (2.70)
Other disorders of urinary system	354 (2.38)	Retention of urine	293 (2.53)
Dsypnea	343 (2.30)	Pneumonia, unspecified organism	244 (2.11)
Other chronic obstructive pulmonary disease	337 (2.26)	Chronic kidney disease (CKD)	227 (1.96)
**Occasional ED Users *(n* = 153,906)**			
**Before the COVID-19 Pandemic (*n* = 84,402)**	**N (%)**	**During the COVID-19 Pandemic (*n* = 69,504)**	**N (%)**
Abdominal and pelvic pain	5306 (6.28)	Abdominal and pelvic pain	4176 (6.00)
Fever of other and unknown origin	4389 (5.20)	Fever of other and unknown origin	3054 (4.39)
Dizziness and giddiness	3052 (3.61)	Dizziness and giddiness	2740 (3.94)
Open wound of wrist, hand, and fingers	2655 (3.14)	Open wound of wrist, hand, and fingers	2402 (3.45)
Superficial injury of head	2339 (2.77)	Pain in throat and chest	1911 (2.74)
Other and unspecified noninfective gastroenteritis and colitis	2269 (2.68)	Superficial injury of head	1719 (2.47)
Other disorders of urinary system	1906 (2.25)	Other and unspecified noninfective gastroenteritis and colitis	1631 (2.34)
Pain in throat and chest	1798 (2.13)	Other disorders of urinary system	1573 (2.26)
Pneumonia, unspecified organism	1746 (2.06)	Superficial injury of knee and lower leg	1542 (2.21)
Injury of unspecified body region	1651 (1.95)	Intracranial injury	1395 (2.00)
